# Neoadjuvant chemoradiation therapy and pathological complete response in rectal cancer

**DOI:** 10.1093/gastro/gov039

**Published:** 2015-08-19

**Authors:** Linda Ferrari, Alessandro Fichera

**Affiliations:** Division of General Surgery, Department of Surgery, University of Washington Medical Center, Seattle, Washington 98195, USA

**Keywords:** rectal cancer, neoadjuvant chemoradiation therapy, pathological complete response

## Abstract

The management of rectal cancer has evolved significantly in the last few decades. Significant improvements in local disease control were achieved in the 1990s, with the introduction of total mesorectal excision and neoadjuvant radiotherapy. Level 1 evidence has shown that, with neoadjuvant chemoradiation therapy (CRT) the rates of local recurrence can be lower than 6% and, as a result, neoadjuvant CRT currently represents the accepted standard of care. This approach has led to reliable tumor down-staging, with 15–27% patients with a pathological complete response (pCR)—defined as no residual cancer found on histological examination of the specimen. Patients who achieve pCR after CRT have better long-term outcomes, less risk of developing local or distal recurrence and improved survival. For all these reasons, sphincter-preserving procedures or organ-preserving options have been suggested, such as local excision of residual tumor or the omission of surgery altogether. Although local recurrence rate has been stable at 5–6% with this multidisciplinary management method, distal recurrence rates for locally-advanced rectal cancers remain in excess of 25% and represent the main cause of death in these patients. For this reason, more recent trials have been looking at the administration of full-dose systemic chemotherapy in the neoadjuvant setting (in order to offer early treatment of disseminated micrometastases, thus improving control of systemic disease) and selective use of radiotherapy only in non-responders or for low rectal tumors smaller than 5 cm.

## Introduction

Multidisciplinary rectal cancer management has become more complex in the last few decades and requires close co-operation between surgeons, medical- and radiation oncologists, and radiologists. Rectal cancer tends to recur locally in the pelvis and to metastasize systemically; therefore, when approaching these patients, attention needs to be directed both at the local and systemic disease.

During the 1970s and 1980s the focus in rectal cancer was on local recurrence, reported as more than 50% after surgery as the only form of treatment. Clinical trials that demonstrated that post-operative radiation decreased rates of local recurrence, and that 5-fluorouracil-adjuvant therapy decreased distal metastases, marked the start of the era of modern rectal cancer treatment [1, 2]. The NCI (National Cancer Institute) consensus statement published in 1990 established that the standard approach to treatment of locally advanced rectal cancer (stages II–III) consisted of surgery, followed by radiation and systemic chemotherapy [3].

Further improvements in local disease control were achieved with the introduction of total mesorectal excision (TME) and neoadjuvant radiotherapy in the 1990s. TME, using sharp dissection along the mesorectal fascia (MRF), has revolutionized the oncological outcomes of patients with resectable rectal cancer, leading to significantly lower local recurrence rates at 10-year follow-up [4–6]. Also the German Rectal Cancer trial randomized 823 patients with cT3–4N+ rectal cancer to either pre-operative or post-operative chemoradiation therapy (CRT) and demonstrated that, at 6%, the rates of local recurrence were lower in the pre-operative CRT group than in the post-operative CRT group (13%; *P* = 0.006) and as a result neoadjuvant CRT is now the standard of care [7, 8].

The extensive use of neoadjuvant CRT has led to an effective tumor down-staging with 15–27% patients with a pathological complete response (pCR)—defined as no residual cancer found on histological examination of the TME specimen [9]. In a recent meta-analysis including 3105 patients, Maas *et al.* demonstrated (i) that the 5-year crude disease survival rate of 484 patients who achieved a pCR after CRT was 83%, compared with 66% for those who did not enjoy pCR (*P* < 0.0001) and (ii) that the 5-year distal metastases-free survival rate was 89% in the pCR group and 75% for non-pCR (*P* < 0.0001) [9].

Patients who achieve pCR after CRT have better long-term outcomes, less propensity to develop local and distal recurrence, and improved survival: for all these reasons, sphincter-preserving procedures or organ-preserving options, such as local excision of residual tumor [10] or omission of surgery altogether, have been suggested [11–14].

There remains the question of how to detect a true pCR; until recently, pCR was determined by surgical resection and pathological evaluation of a specimen. More recently it has been shown that endoscopic and radiological assessment following CRT can reliably identify patients who achieved a clinical complete response (cCR), and a ‘wait and see‘ approach, with close follow-up, has been proposed.

Although local recurrence rate has been stable at 5–6% with this multidisciplinary management strategy, distal recurrence rates for locally-advanced rectal cancers are still in excess of 25% and now represent the main cause of death in these patients. For this reason, more recent trials have been looking at the administration of full-dose systemic chemotherapy in the neoadjuvant setting, to offer early treatment of disseminated micrometastases, thus improving control of systemic disease [15–17].

## Selecting patients for neoadjuvant therapy

Rectal cancer staging has to provide information about locoregional and systemic disease. Modern multi-detector computed tomography (CT) scanners are very reliable in ruling out distal disease, while endorectal ultrasound (ERUS) and magnetic resonance imaging (MRI) provide different and complementary information about local staging and involvement of sphincteric and pelvic structures [18]. ERUS has a short focal range and can clearly identify different layers of the bowel wall, thus providing essential information in early rectal cancer [19]. MRI is the ’gold standard’ for assessing the relationship between rectal cancer and pelvic anatomical structure (particularly in the MRF), which is considered a critical landmark in the pre-operative evaluation of these patients [20–25].

The prospective, observational Magnetic Resonance Imaging and Rectal Cancer European Equivalence (MERCURY) trial evaluated the accuracy of MRI in predicting a curative resection of rectal cancer and reported a specificity of 92% in predicting a negative circumferential margin (CRM) [22]. The 5-year follow-up results of the MERCURY study demonstrated that a clear circumferential margin, as evaluated by high-resolution pelvic MRI (“CRM clear MRI”), was the only pre-operative parameter that was significant for overall survival (OS), disease-free survival (DFS) and local recurrence [23]. The 5-year OS was 62.2% in patients with MRI-clear CRM, compared with 42.2% in patients with CRM involved MRI (*P* < 0.01); the 5-year disease-free survival was 67.2% for MRI-clear CRM, compared with 47.3% for MRI-involved CRM (*P* = 0.05); the local recurrence hazard ratio was 3.50 for MRI-involved CRM (*P* < 0.05). In the multivariate analysis, MRI involvement of the CRM was the only significant parameter for local recurrence and survival, and significantly associated with development of distal metastatic disease (25% during 5-year follow-up).

The MERCURY study was also able to identify a group of rectal cancer patients with “good prognosis” characterized by (i) MRI-clear CRM, (ii) no extramural venous invasion, (iii) T2/T3a/T3b, (iv) less than 5 mm spread from the *muscularis propria*, (v) no involvement of the intersphincteric plane, regardless of the MRI N stage [24]. These patients underwent surgery as the only treatment and 85% had 5-year DFS and only 3% local recurrence. In addition, the recent MERCURY II study evaluated risk factors for pathological CRM rates in low rectal cancer assessment (distal margin at or below 6.0 cm from anal verge) by high resolution MRI [25]. They compared MRI findings with pathology reports and showed that CRM and/or intersphincteric involvement found on MRI increased 5-fold pathological circumferential resection margin rate compared with patients without involvement of these structures (odds ratio = 5.5; 95% confidence interval, 2.3–13.3). In addition, if both of these margins were clear on MRI for re-staging (after neoadjuvant CRT), the concordance with pathological examination was 100%. They concluded that MRI should drive treatment, even for low-rectal cancer, with a good prognostic value.

**Figure 1.**
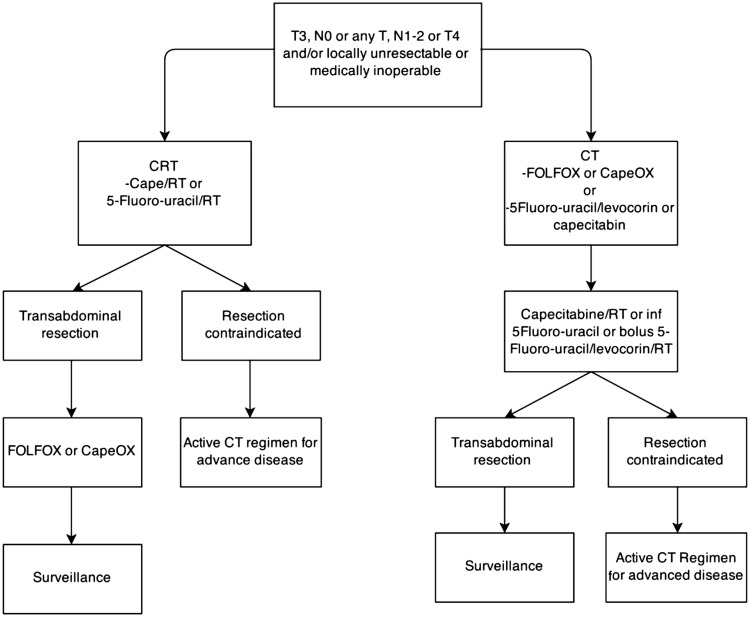
Management of rectal cancer in the USA according to NCCN guidelines [26] CRT = chemoradiotherapy; CT = chemotherapy; RT = radiotherapy

**Figure 2.**
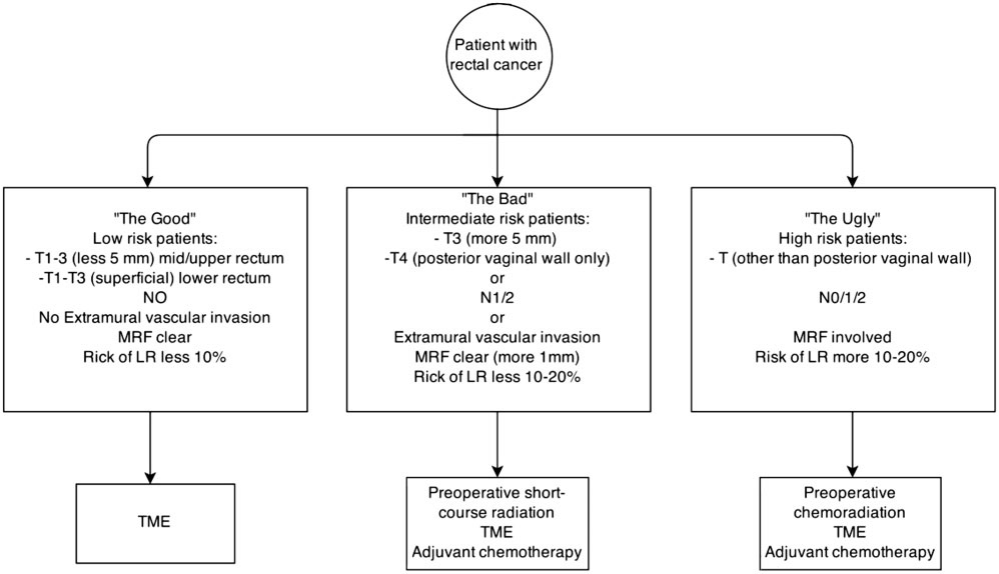
European management of rectal cancer [27–29] LR = local recurrence; MRF = mesorectal fascia; TME = total mesorectal excision

The information provided by MRI is used in different ways in different parts of the world; in the USA, for example, treatment of rectal cancer is based on clinical tumor, node, metastasis (TNM) staging system. According to National Comprehensive Cancer Network (NCCN) guidelines, patients with locally-advanced rectal cancer (T3N0 or any T with N1–N2) are managed with long-course CRT, followed by TME and eventually followed by adjuvant therapy (Figure 1) [26]. In Europe and Scandinavia, based on the data reported above and prospective randomized trials, rectal cancer patients are stratified according to MRI findings into three risk-related grades: (i) low risk (“the good”), treated with surgery alone, (ii) intermediate risk (“the bad”), treated with pre-operative short-course radiation plus TME and, eventually, adjuvant chemotherapy and (iii) high risk (“the ugly”) managed by long-course pre-operative CRT followed by TME and adjuvant chemotherapy (Figure 2) [27–29].

## Neoadjuvant radiotherapy and chemoradiotherapy

Local disease control with low-recurrence rates is achieved either with pre-operative short course radiation therapy (SCRT) (5 Gy per day; total dose of 25 Gy) or long-course fractionated radiation (1.8–2.0 Gy per day; total dose of 45–50 Gy) combined with sensitizing fluoropyrimidine, followed by surgery [7, 8, 30–35]. Short-course radiation does not include administration of sensitizing chemotherapy and surgery is usually performed within 7 days, while long-course treatment involves chemotherapy and surgery is performed within 6–8 weeks. Short- and long-course neoadjuvant therapy has been compared in two randomized trials [36, 37].

The Polish trial compared SCRT and long-course CRT in 312 patients with clinical T3–T4 tumors, to evaluate the difference in sphincter preservation rates between these two treatments [36]. The proportion of sphincter preservation surgery was similar in each group: 61% in the SCRT group *vs.* 58% in the CRT group; in addition they found higher rates of complete response in the CRT group (16% *vs*.** 1% in the SCRT group) with involvement of the circumferential margin also greater in the SCRT group (12.9% *vs*.** 4.4% in the CRT group; *P* = 0.0017).

The Trans-Tasman Radiation Oncology Group analysed 326 patients with clinical T3N0-2M0 tumors within 12 cm of the anal verge, randomized to either SCRT or long-course CRT followed by surgery after 4–6 weeks, then by adjuvant chemotherapy after a further 4 weeks [37]. Local recurrence rate was higher in the SCRT group than with CRT (7.5% *vs.* 4.4%), although not statistically significant (*P* = 0.24); unfortunately more patients in the short-course group had distal tumors (lower than 5 cm from the anal verge) than in the CRT group (30% *vs*.** 19%) and this could, in part, explain the difference. In reality the controversy between these two modalities is still unsettled, even if long-course CRT offers higher rates of pCR.

In of the matter of sensitizing chemoradiation, the NSABP R04 study demonstrated that intravenous 5-fluoruracil (5-FU) or the equivalent pro-drug capecitabine (converted to fluorouracil by intracellular thymidine phosphorylase) have equivalent effects. The study randomized 1608 patients to 5-FU or capecitabine and found no differences in terms of pCR, early local recurrence or DFS [38]. This study also compared the addition of oxaliplatin to 5-FU-based chemoradiation, with no incremental benefits found. Similar results were obtained from Gerard *et al*.** in the Actions Concertees dans les Cancers Colorectaux et Digestifs (ACCORD) 12/0405-Prodige2 trial, where adding oxaliplatin once per week to fluoropyrimidine-based CRT resulted in a worse therapeutic ratio and higher toxicity [39].

## Improving pathological complete response: increased surgical interval and consolidation chemotherapy

Neoadjuvant CRT in rectal cancer is associated with improved local control, and results in pCR in 15–27% of cases [9]. Park *et al.* retrospectively studied 725 patients with locally advanced rectal cancer, treated with neoadjuvant radio-chemotherapy (RCT) followed by radical surgery, to assess how oncological outcomes are associated with degree of pathological response [40]. Pathological classification was ypT0N0 in 131 patients (18.1%), ypT1–2N0 in 210 (29.0%), ypT3–4N0 in 164 (22.6%), and any ypT with N+ in 210 (29.0%). Patients were divided into three categories based on response rate: ypT0N0 was classified as complete response (CR), ypT1–2N0 was classified as intermediate response (IR) and ypT3–4N0 or ypTanyN+ were classified as poor response (PR). The median follow-up was 65 months and was shorter in the PR group than in the other response groups (*P* < 0.001), largely because of cancer-related mortality. The 5-year overall survival rates for the CR, IR and PR groups were 93.4%, 87% and 77.3%, respectively (*P* = 0.002); the 5-year recurrence-free survival (RFS) rates were 90.5%, 78.7% and 58.5%, respectively (*P* < 0.001). The researchers concluded that response to neoadjuvant treatment is an early surrogate marker and correlates with oncological outcomes, and these results can drive novel treatment strategy and approaches.

The discovery that patients with a pCR have a more favorable prognosis than those with residual disease leads investigators to believe that the achievement of pCR should be considered to be a goal in rectal cancer and that strategies to obtain a pCR should be implemented.

One approach is to lengthen the interval between CRT and surgery, because it has been proven that pCR is time-dependent [41–44]. Tulchinsky *et al*.** studied the interval between RCT and surgery in 132 patients with locally advanced rectal cancer [41]. Patients who underwent surgery more than 7 weeks after RCT had a pCR rate of 35%, compared with 17% for patients operated on less than 7 weeks after RCT (*P* = 0.03). Kalady *et al*.** reported 31% pCR in patients operated on more than 8 weeks after RCT, compared with 16% receiving surgery within 8 weeks [42]. Zeng *et al*.** looking at 233 patients, finding that perioperative complications were not influenced by a longer interval and, in addition, patients operated on more than 7 weeks after CRT had a higher pCR rate (27.1% *vs*.** 15.3%; *P* = 0.029) and a decreased rate of circumferential resection margin involvement (1.6% *vs*.** 8.1%; *P* = 0.020). After a median follow-up of 42 months, the 3-year local recurrence rate was 12.9% in the short-interval group (less than 7 weeks) *vs.* 4.8% in the long-interval group (*P* = 0.025) [44].

Another strategy for achieving pCR is to administer additional chemotherapy (known as ‘consolidation chemotherapy’) during the waiting period between CRT and surgery [45–49]. This approach was originally described by Habr-Gama *et al*.*,* who enrolled 70 patients with rectal cancer located within 7 cm from the anal verge and stage cT2–T4 or cN1–N2 [45, 46]. Patients underwent neoadjuvant CRT consisting of 54 Gy of radiation and, concurrently, three cycles of bolus 5-FU/leukovorin. Following completion of CRT, patients received three additional, identical cycles of chemotherapy every 3 weeks (over 6 weeks in total). Tumor reassessment was performed at 6 weeks and 10 weeks after treatment by endoscopic examination, plus MRI or ERUS. Their definition of sustained cCR was patients who achieved cCR and maintained it for the followed 12 months. Thirty-nine patients (51%) achieved sustained cCR that never required surgery for locally recurrent cancer after a median follow-up of 56 months; among them, 3-year OS and DFS were 94% and 75%, respectively, with a median follow-up of 53 months.

Garcia-Aguilar *et al*.** reported the early results from 127 patients in a multicenter, non-randomized trial and evaluated the improvement in pCR resulting from increasing the waiting period between RCT and surgery and administering additional chemotherapy during this interval [48]. Rectal cancer clinical stage II (T3–4N0) and III (any T, N1–2) and all patients received long-course CRT were reassessed by proctoscopic examination 4 weeks after completing treatment. Patients with clinical partial response or clinical complete response received two additional cycles of FOLFOX-6 (Folinic acid [leucovorin], Fluorouracil [5-FU], oxaliplatin [eloxatin]) and had surgery 3–5 weeks after the last cycle and were referred to as surgical group 2 (SG2); these were compared with standard of care (radiochemiotherapy followed by surgery), identified as surgical group 1 (SG1). The average times between CRT and surgery were 6 weeks for SG1 and 11 weeks for SG2. Better oncological outcomes were achieved in SG2, in terms of overall pathological response (*P* = 0.02178) and pathological T-stage (*P* = 0.0008); pCR was more common in SG2 (25%) than in SG1 (18%) but the difference did not reach statistical significance. Incidentally, the surgeons reported worse pelvic floor fibrosis in the SG2 group, but without increased surgical difficulties or complications.

The final results of this study were reported as part of the TIMING (Timing of Rectal Cancer Response to Chemoradiation Consortium) trial (NCT00335816; timing of rectal cancer response to chemotherapy trial) [18, 48, 49]. The study showed that delivering two, four, or six cycles of FOLFOX after CRT in patients with locally advanced rectal cancer increased the pCR rates to 25%, 30% and 38%, respectively, (while CRT alone achieved only 18%), without affecting surgical complications and with good compliance; in fact 80% of patients completed consolidation chemotherapy without interruption.

These studies suggest that administering full-course chemotherapy in the neoadjuvant setting offers higher pCR rates, with good compliance and acceptable side-effects and surgical complications.

## Decreasing rates of systemic recurrence: consolidation/Induction chemotherapy

Since the introduction and widespread utilization of TME, local recurrence rates have significantly decreased, also due to the use of pre-operative radiotherapy or CRT; however these modalities have no effect on distal metastasis. Radiotherapy is effective locally but chemotherapy with fluoropyrimidine alone is not adequate for systemic treatment, either because is administered as single agent—not as effective as combination therapy—or because dosing is reduced for concurrent administration of radiotherapy. Local recurrence rates are stable at 5–6% with neoadjuvant CRT followed by TME; however the rate of distal metastasis remains stable at about 25%. For this reason, patients with locally advanced rectal cancer after neoadjuvant CRT and TME should receive post-operative adjuvant chemotherapy based on the clinical pre-treatment staging and independent of response to chemotherapy [50]. Similarly to patients with colon cancer, these patients receive fluorouracil or capecitabine plus oxaliplatin-based adjuvant chemotherapy [50].

Contrary to these recommendations, approximately 27% of patients with locally advanced rectal cancer never start adjuvant therapy and less than 50% receive full-dose chemotherapy [51–54]. Khrizman *et al*.** found that the most frequent reason why patients do not receive chemotherapy after surgery is the presence of comorbid conditions and, even when chemotherapy was offered, patient refusal is the most frequent reason for lack of compliance [54]. Furthermore a systematic review of 10 studies including 15 410 patients has demonstrated a 14% decrease in overall survival for every 4 weeks’ delay in administration of adjuvant therapy; beyond 12 weeks after surgery, it is unclear if adjuvant chemotherapy still offers any benefits [55].

One strategy to facilitate administration of full-dose chemotherapy is induction chemotherapy given before CRT, with the following sequence: induction chemotherapy followed by CRT, followed by TME and eventually additional adjuvant chemotherapy.

The efficacy of induction chemotherapy has been proven by several studies [56–61]. One option is to split adjuvant chemotherapy by delivering a limited number of cycles before CRT and then to administer the remaining cycles in the adjuvant setting, or to deliver full-course systemic chemotherapy before CRT and surgery. Chua *et al*.** enrolled 105 patients, classified as poor prognosis rectal cancer defined by MRI, to the following protocol: four cycles of oxaliplatin and capecitabine, followed by 6 weeks of CRT with oral capecitabine; after 6 weeks they underwent TME and after recovery from the operation another 12 weeks of capecitabine monotherapy [57]. The patients were staged by MRI after completion of induction chemotherapy and after CRT. Radiological complete response was 3% after induction chemotherapy and 14% after CRT; radiological response rates after induction chemotherapy and CRT were 74% and 89%, respectively. At surgery, pCR was 20%. The 3-year progression-free survival and overall survival rates were 68% (95% CI: 59–77%) and 83% (95% CI: 76–91%), respectively.

Schou *et al*.** applied a similar protocol to 84 patients with T4, T3N+ or T3 tumors involving—or within <1 mm of—the MRF. They received two cycles of CapeOx (capecitabine and oxaliplatin), followed by radiotherapy with concurrent capecitabine, and TME 6 weeks after completion of CRT [59]. Pathological complete response was seen in 23% of patients; in addition 5-year DFS and OS were 63% (95% CI: 52.2–73.7%) and 67% (95% CI: 56.1–77.3%), respectively. More recently, Cercek *et al*.** enrolled 61 patients with stage II–III rectal cancer. Thirty patients received all their eight cycles of FOLFOX ’up front’; the others receiving some of theirs before surgery and some after; all patients completed chemoradiation without interruption [61]. Among 61 patients receiving initial FOLFOX, 22 (36%) enjoyed either pCR (*n* = 13) or cCR (*n* = 9); these latter nine patients were managed without surgery. Of the 49 patients who underwent TME, all had R0 resections and 23 (47%) had tumor response greater than 90%, including 13 (27%) who went on to pCR. Among 28 patients who received all eight cycles of ‘up front’ FOLFOX, eight achieved pCR (29%) and three cCR (11%).

Several hypothetical advantages of chemotherapy administered in the neoadjuvant setting are worth mentioning: (i) this approach allows delivery of chemotherapy to a well-vascularized primary tumor in patients who are not recovering from a major operation; (ii) neoadjuvant chemotherapy can address distal micrometastatic disease early in the course and (iii) it permits a longer interval between CRT and surgery, thus increasing pCR rates, resulting in an overall survival rate of 83–95% in patients with pCR [9, 62].

## Neoadjuvant chemotherapy without radiotherapy: does everybody need combined modality therapy?

Risk of local recurrence in locally advanced rectal cancer is dependent on tumor stage, and distance from the anal verge and MRF.

Upper rectal tumors away from the MRF have low risk of local recurrence, even if treated with TME alone. Gunderson *et al*.** showed that patients with intermediate-risk rectal cancer (T1–2N1 and T3N0) had similar 5-year OS with surgery followed by adjuvant chemotherapy or with trimodal treatment regimens (radiotherapy + bolus chemotherapy; radiotherapy + protracted venous infusion chemotherapy; radiotherapy + bolus chemotherapy) [63, 64]. For surgery + chemotherapy, 5-year OS was 85% for T1–2N1 lesions and 84% for T3N0 lesions and, for trimodal treatment, 83% and 74%, respectively. Five-year DFS for surgery + chemotherapy was 78% for T1–2N1 lesions and 69% for T3N0 lesions; for trimodal therapy, 5-year DFS ranged from 75–78% for patients with T1–2N1 lesions and from 63–75% for those with T3N0 lesions.

Another study from Massachusetts General Hospital investigated indications for adjuvant radiotherapy and chemotherapy in patients with T3N0 lesions [65]. The researchers found that patients with favorable histological features (well- or moderately well-differentiated tumors; invasion less than 2 mm into the perirectal fascia; no evidence of lymphatic or venous involvement) had significantly better disease control and recurrence-free survival than those with unfavorable lesions. In patients with favorable features, 10-year actuarial rates of local control and RFS were 95% and 87%, respectively, against 71% and 55% for patients with unfavorable features. The authors concluded that patients with surgically resected T3N0 lesions with favorable histological features and a negative radial resection margin can be treated with surgery alone, without adjuvant chemotherapy.

Considering that radiotherapy is associated with sexual and urinary dysfunction and proctitis [66, 67], it has been queried as to whether some patients can be safely treated with chemotherapy alone without an increase in local recurrences.

A recent European multicenter trial has investigated the selective use of radiation among 46 patients with T3 rectal adenocarcinoma located in the middle third with clear MRF, as selected by MRI [15]. Patients received four cycles of neoadjuvant capecitabine and oxaliplatin, combined with bevacizumab (final cycle without bevacizumab) before TME. In cases of progression, pre-operative CRT was offered. No progression was detected; overall response rate was 78% (*n* = 36; 95% CI: 63–89%) and pCR was observed in nine patients (20%; 95% CI: 9–33%); 2-year DFS rate was 75% (95% CI: 60–85%) and 2-year local relapse rate was 2% (95% CI: 0–11%).

A similar protocol was studied by Schrag *et al*.** in 32 patients, who received four cycles of modified FOLFOX with bevacizumab [16]. In cases of disease progression or side-effects of chemotherapy, CRT was available for ‘salvage‘. Only two patients received CRT due to intolerance to bevacizumab. A R0 resection was performed in all patients and pCR was achieved in 8 of 32 patients (25%); 4-year DFS was 84% (95% CI: 67–94%) and no local recurrences were noted.

**Figure 3.**
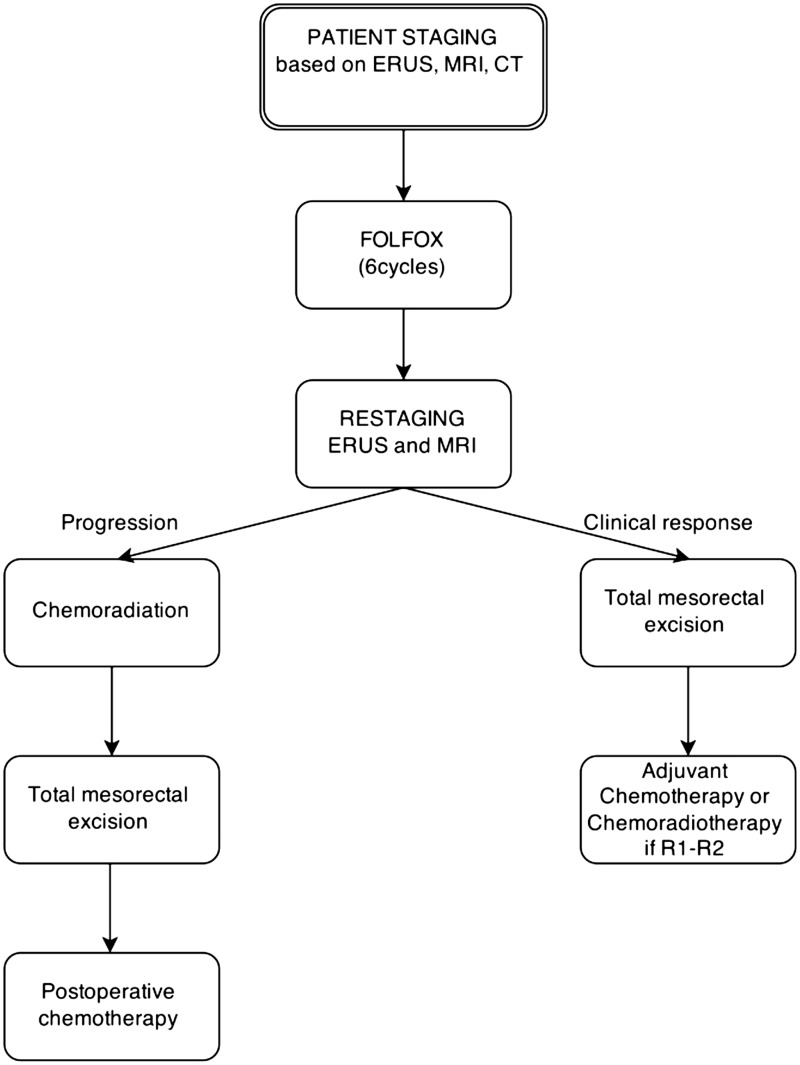
The PROSPECT trial CT = computed tomography; ERUS = endorectal ultrsound; MRI = magnetic resonance imaging

This study provided the preliminary data to design the Phase II/III PROSPECT (Preoperative Radiation or Selective Preoperative Radiation and Evaluation before Chemotherapy and Total Mesorectal Excision) trial (chemotherapy alone or chemotherapy plus radiation therapy in treating patients with locally advanced rectal cancer undergoing sphincter preserving surgery). Patients can be included if the cancer is at least 5 cm from the anal verge, cT stage <cT4, nodal stage <cN2, and there is no disease within 3 mm of the MRF. The objective of this trial is to determine whether pelvic radiotherapy can be used selectively, based on patient response to neoadjuvant FOLFOX (Figure 3).

## Non-surgical management of rectal cancer

In 2004, Habr-Gama *et al*.** reported their experiences with 265 patients with cT2–T4N0/N+ rectal adenocarcinoma, who underwent pre-operative CRT consisting of 5040 cGy over 6 weeks, and 5-FU/leukovorin administered for three consecutive days of CRT [11]. All patients were re-evaluated after 8 weeks by endoscopy and biopsy. They defined cCR as absence of residual ulceration, stenosis, or mass within the rectum during digital rectal examination and proctoscopy 8 weeks after CRT. Radiological confirmation of cCR was obtained, together with a carcinoembryonic antigen (CEA) level. Patients with cCR were managed without surgery by monthly physical and digital rectal examination, proctoscopy, biopsies and CEA level for the first year. This was carried out every 2 months in the second year and every 6 months in the third one. CT of the chest-abdomen-pelvis was carried out every 6 months in the first year (Table 1). Seventy-one patients had a cCR and were enrolled in the non-surgical management 8 weeks after completion of CRT; of these patients 49 (69%) had T3 lesions, 14 (19.7%) had T2 lesions and 8 (11%) had T4 lesions; only 16 (23%) had radiological evidence of N+ lesions. Among these 71 patients, the 5-year OS was 100% and DFS 92%, compared with 88% and 83%, respectively, among patients in the control group, who did not achieve cCR and went to TME after CRT. Only two patients (2.8%) developed endoluminal recurrence after 56 and 64 months of CRT and were treated with local excision and brachytherapy.

**Table 1.** Follow-up schedule for patients who achieved clinical complete response (cCR) enrolled in the ‘watch and wait’ study by Habr-Gama *et al.* [11]

**Year 1**
Every month: DRE, endoscopy, CEA
Every 6 months: CT for distal staging
**Year 2**
Every 2 months: DRE, endoscopy, CEA
Every 12 months: CT for distal staging
**Year 3**
Every 6 months: DRE, endoscopy, CEA
Every 12 months: CT for distal staging

CEA = carcinoembryonic antigen; CT = computed tomography; DRE = digital rectal examination

The same authors published a larger series of 361 patients with cT2–4 tumors, 99 (27%) achieved sustained cCR, defined as clinical complete response maintained for more than 12 months [12]. Only five patients (5%) developed endorectal recurrence after 18, 43, 56, 64 and 79 months of follow-up, respectively, while seven patients (7%) developed systemic recurrence. The 5-year DFS was 85% and OS was 93% and the authors concluded that ‘wait-and-see’ management of rectal cancer for cCR can be safety recommended, since survival in these patients is affected primarily by systemic failure.

In 2011, Maas *et al*.** published a retrospective study of 21 patients with cCR, who had been managed by a ‘watch and wait‘ approach [14]. They recruited 192 patients with cT1–3N0-2 who were treated with CRT of 5040 cGy over 28 fractions with concurrent capecitabine. In this study, the criteria for cCR were more strict than in the Habr-Gama study and, based on MRI and endoscopic assessment, were defined as (i) no residual tumor on the MRI and only fibrosis present, (ii) no suspicious lymph node on MRI, (iii) only residual ulcer or scar at endoscopy, (iv) negative biopsies from the scar, ulcer or former tumor location and (v) no palpable lesion on digital rectal examination. The 21 patients who met these criteria underwent close follow-up consisting of digital rectal examination, MRI and endoscopy every 3 months for the first year and every 6 months for the second and third years, CT scan of chest and abdomen every 6 months in the first year and every 12 months in the second and third years. In addition, CEA levels were measured every 3 months for the first 3 years (Table 2). The median follow-up was 25 months and only one patient developed endoluminal recurrence, undergoing transanal endoscopic microsurgical excision of the mass. In this series, the control group comprised the 20 patients who were found to have pCR after TME. The 2-year OS and DFS in the ‘watch and wait’ group were 100% and 89%, respectively; in the control group, 2-year OS and DFS were 91% and 93%, respectively. The results were similar in each group and the authors concluded that non-surgical management can be safely applied in selected patients.

**Table 2.** Follow-up schedule for patients who achieved clinical complete response (cCR) enrolled in the ‘watch and wait’” study by Maas *et al.* [14]

**Year 1**
Every 3 months: CEA, DRE, endoscopy and MRI
Every 6 months: CT for distal staging
**Years 2–3**
Every 3 months: CEA
Every 6 months: DRE, endoscopy, MRI
Every 12 months: CT for distal staging
**Years 4–5**
Every 6 months: CEA, DRE, endoscopy and MRI
Every 12 months: CT for distal staging

CEA = carcinoembryonic antigen; CT = computed tomography; DRE = digital rectal examination; MRI = magnetic resonance imaging

More recently, Smith *et al*.** published the results from a retrospective study; they compared the outcomes of 32 stage I–III rectal cancer patients with cCR after CRT, who were treated non-surgically, against 57 patients with pCR after radical rectal resection [13]. The cCR was documented by non-palpable lesion on digital rectal examination and endoscopy, showing no visible pathology other than a flat scar. The close follow-up consisted of physical examinations and flexible sigmoidoscopies every 3 months for the first year and every 4 months the second year and every 6 months thereafter; ERUS and MRI were not routinely used. The median follow-up was 28 months; six patients in the non-surgical management group developed local recurrence and three of them also developed distal metastases; all these six recurrences were controlled by salvage rectal resection and TME, with no further local recurrences of disease at median follow-up of 17 months. The non-surgical management group had a higher rate of local recurrences (2-year actual rate of local recurrence was 21% *vs*.** 0%; *P* = 0.001); however the 2-year rates of distal recurrence (8% *vs*.** 2%; *P* = 0.30), 2-year DFS (88% *vs*.** 98%; *P* = 0.27) and OS (97% *vs*.** 100%; *P* = 0.56) were similar.

These studies showed that, with accurate identification of cCR and close follow-up, patients can be monitored, thus avoiding immediate TME and still have good oncological outcomes (Table 3). This conservative approach is particular attractive for elderly and medically unfit patients; in fact, Smith *et al*.** demonstrated that a ‘watch and wait’ approach improved survival at a 1-year time point in all patients and increased incrementally with age and comorbidities [68]. They categorized absolute survival into three groups of patients: (i) fit 60-year old males (f60), (ii) fit 80-year old males (f80) and (iii) 80-year-old males with significant comorbidities (c80). The improvement in OS in the three groups was as follows: (i) f60 2% (95% CI: 0.9–3.1%), (ii) f80 10.1% (95% CI: 7.9–12.6%), (iii) c80 13.5% (95% CI: 10.9–16.2%). For elderly patients the risk of death within 6 months after surgery is 2–3 times as great as at 1 month [69].

**Table 3.** Outcomes of patients who achieved clinical complete response (cCR) and underwent ‘watch and wait’ management

Studies	No. of patients	Follow-up (months)	Overall survival (%)	Disease-free survival (%)
Habr-Gama *et al.* [12]	122	60	5-year: 93	5-year: 85
Smith *et al.* [13]	32	28	2-year: 97	2-year: 88
Maas *et al.* [14]	21	5	2-year: 100	2-year: 89

## Assessment of complete response: how to Identify the true response

The feasibility of the ‘watch and wait’ approach is strictly dependent on the accurate identification of patients who achieved cCR after CRT, which represents the critical aspect of this management [70].

Digital rectal examination is an important diagnostic maneuver for assessing tumor response to treatment. A cCR is defined as the absence of any irregularity of the rectal wall and the surface has to be regular and smooth [71]. Endoscopic assessment is also very important, and permits evaluation of mucosal characteristics: no residual mass, ulceration or stenosis should be detected, but only whitening of the mucosa and telangiectasias [71]. During rigid proctoscopy the response can be evaluated by biopsies, even though the results should be interpreted with caution; in fact, according to a recent study, after CRT, cancer cells can be detected deeper within the layers of the rectal wall even if no cancer cells are present in the mucosa [72]; for this reason superficial biopsies could lead to false-negative results and, if a biopsy is indicated, it should include the deeper layers.

The desirability of a full-thickness local excision (FTLE)—to provide pathological confirmation of a complete primary tumor response (ypT0), eliminating the risks of having microscopic residual cancer foci behind in the setting of cCR—remains an open question. The drawbacks of this strategy are several: the healing of the rectal wall after FTLE following CRT takes time and is associated with significant side-effects and pain, particularly when close to the anal verge. In addition, in patients who have undergone FTLE, the detection of local relapse during follow-up is limited and delayed by scarring and residual mucosal abnormalities. Finally, in cases of radical surgery due to recurrence after FTLE, scarring in the mesorectum makes the dissection cumbersome, reducing the ability to perform a sphincter-preserving operation. For all these reasons follow-up is facilitated by preservation of rectal wall integrity [73, 74]. Moreover, FTLE is not able to sample local lymph nodes which, even in the case of a yT0, could still be positive in 5–10% cases (ypT0N1) [9].

Regarding imaging techniques, high-frequency three-dimensional endorectal ultrasound (3D-ERUS) can identify residual tumors and complete response with a high degree of accuracy [75, 76], and can help in the choice of surgical approach or non-surgical management. The appearance of normal anatomy of the rectal wall under ultrasound, at the former tumor site after CRT, can predict complete response with good accuracy [75], even though the result should be interpreted with care and needs to be integrated with the findings of other imaging and endoscopic examinations.

Traditional MRI is important to the initial stageing of rectal cancer patients [20–25] but it is less important to re-stage patients after neoadjuvant treatments [77, 78]. A recent meta-analysis, including 33 studies and 1556 patients, on the use of MRI imaging for re-staging locally advanced rectal cancer after CRT, showed a mean sensitivity of 50% and specificity of 91%. In the subgroup analysis, MRI demonstrated 19% sensitivity and 94% specificity for identifying pT0 disease but cannot predict pCR [77]. This is due to the inability of MRI to differentiate between tumor tissue and either post-therapeutic fibrosis or edema following neoadjuvant CRT.

After introducing functional MRI imaging, such as diffusion-weighted MRI (DW-MRI) and dynamic contrast-enhanced MRI (DCE-MRI), sensitivity and specificity in detecting pCR are improved [78]. DW-MRI increased accuracy in detecting viable tumor cells from 64–76% to 86–90% [79]. Recently Lambrecht *et al*.** studied diffusion data from 20 patients with rectal cancer before and after neoadjuvant CRT and reported a sensitivity of 100% and specificity of 93–100% for pCR [80]. A multicenter study demonstrated that the addition of DWI to T2-weighted imaging improve detection of complete response after therapy, with a sensitivity of 52–64% (compared with 0–40% for T2-weighted imaging alone) and specificity of 89–98% [81].

Dynamic contrast MRI, or perfusion imaging, allows the evaluation of the local microcirculation and capillary permeability in the tumor tissue, by measuring the changes in signal intensity over time and after injection of paramagnetic contrast agent; the resultant changes in signal intensity can be evaluated semi-quantitatively. Lim *et al*.** reported that high values of permeability and/or flow K^(trans)^, into the tumor before starting the therapy correlates with good response to treatment [82]. Gollub *et al*.** conducted a similar study of patients receiving chemotherapy alone and coupled with an anti-angiogenic agent (bevacizumab), and shown that the post-therapeutic value of K^trans^ differed considerably between patients with and without pCR.

For the nodal stage, MRI has a mean sensitivity of 77% and specificity of 60% [77]. Lambregts *et al*.** studied the use of gadofosveset—an albumin-bound gadolinium chelate approved for magnetic resonance angiography—and they reported that, compared with T2-weighted imaging alone, imaging with gadofosveset increased sensitivity (80% *vs*.** 76%) and specificity (97% *vs*.** 82%) [84, 85].

Even positron emission tomography–computed tomography (PET-CT) can be used to assess tumor response after CRT; it allows the visual identification of the uptake of fluoro-2-deoxy-D-glucose (FDG) within the area of the rectal wall or within the mesorectum and can estimate the metabolism profile. A prospective study has looked at complete and incomplete responses and found a good accuracy of 85% [86]. In a prospective study, Guillem *et al*.** evaluated the identification of complete response after pre-operative CRT; they enrolled 121 patients with locally advanced rectal cancer and obtained PET and CT images before and after CRT [87]. In total, 26 patients (21%) had pCR after CRT, but only 54% of the pCR patients were classified as having a cCR on pre-operative PET scan and only 19% on CT; the authors concluded that neither PET nor CT scan alone can predict pCR.

Pre- and post-treatment CEA levels are important in assessing patients with rectal cancer. In a recent retrospective study, Kleiman *et al.* evaluated 141 patients who underwent CRT and divided them into two groups—pCR or not—based on final pathology [88]. CEA levels were measured before and after treatment, 19 patients (13.5%) achieved pCR, while 122 (86.5%) did not. Pre-treatment CEA levels were similar in both groups (2.75 *vs*.** 4.5 µg/L; *P* = 0.65), post-treatment CEA levels were lower in patients with pCR (1.7 *vs*.** 2.4 µg/L; *P* < 0.01). Multivariate analyses demonstrated that post-treatment CEA level was an independent predictor of pCR (OR 1.74; 95% CI: 1.06–3.81) and normalization of CEA from initially elevated level was a significant predictor of pCR (OR 64.8; 95% CI: 2.53–183.71).

In conclusion, cCR can be assessed only by a combination of physical examination, endoscopic and laboratory examinations and imaging. Patients with a cCR managed non-surgically should be closely followed up.

## Conclusions

Neoadjuvant CRT has significantly improved over the last 20 years and it is very effective in reducing local recurrence of tumors. Unfortunately, distal disease still occurs in more than 25% of patients during follow-up and represents the primary cause of death. Induction/consolidation chemotherapy and chemotherapy alone are new strategies shown to increase pCR rates, the ultimate goal of neoadjuvant treatment. Achieving pCR correlates with improved DFS and is considered a good prognostic marker. Clinical strategies to detect pCR and safe implementation of a ‘watch and wait‘ strategy are being developed, for an organ-preserving approach to the disease.

**Conflict of interest statement:** none declared.
